# Evaluation of Buriti (*Mauritia flexuosa* L.) Oil as an Additive for Carbohydrate-Based Biodegradable Films

**DOI:** 10.3390/foods14244330

**Published:** 2025-12-16

**Authors:** Bárbara L. S. Freitas, Noemi P. Almeida, Felipe F. Haddad, Leandro S. Oliveira, Adriana S. Franca

**Affiliations:** 1 PPGCA, Departamento de Alimentos, Faculdade de Farmácia, Universidade Federal de Minas Gerais, Campos Pampulha, Av. Antônio Carlos, 6627, Belo Horizonte 31270-901, MG, Brazil; barbaraluiza.s.f@gmail.com (B.L.S.F.); noemipalmeida@gmail.com (N.P.A.); leandro@demec.ufmg.br (L.S.O.); 2 DCA, Escola de Ciências Agrárias, Universidade Federal de Lavras, Lavras 37203-202, MG, Brazil; felipe.haddad@ufla.br; 3 DEMEC, Escola de Engenharia, Universidade Federal de Minas Gerais, Campos Pampulha, Av. Antônio Carlos, 6627, Belo Horizonte 31270-901, MG, Brazil

**Keywords:** bioplastics, by-products, galactomannans, starch

## Abstract

Recent studies have focused on the development of food packaging films based on biopolymers, with polysaccharides being at the forefront due to their abundant availability in food and agricultural by-products. Therefore, it was the aim of this work to prepare and characterize conjugated biopolymeric films using starch, galactomannans and buriti oil (BO), with the latter acting as a partial or integral replacement for glycerol as a plasticizer. The addition of BO to either the starch or the conjugated starch–galactomannan film formulations led to distinct interactions between the components and consequently to films with distinct properties. The addition of both BO and galactomannan to starch hindered retrogradation, characterized by a diminished degree of crystallinity in comparison to the film containing only starch, attesting the stabilization of the starch molecular structures in its interaction with galactomannan molecules and with the emulsified BO. The analyses of films’ mechanical properties demonstrated that the added BO did not act as a plasticizer, leading to increased tensile strength and elastic modulus and decreased elongation at break in all formulations. Overall, the films containing starch presented lower mechanical resistance than the ones based on galactomannan. All formulations led to biodegradable films, with those with BO taking longer to degrade.

## 1. Introduction

Plastic food packages function as physical and chemical barriers that mitigate the impact of environmental factors that may contribute to food spoilage, while concurrently enhancing microbial safety, maintaining physicochemical quality attributes, and prolonging product shelf life [1]. However, the environmental challenges posed by the improper disposal of plastics have prompted studies on the development of biodegradable and renewable films for food applications. Also, agricultural and food wastes or by-products are rich sources of biopolymers (polysaccharides, proteins and others) that have been extensively studied, aiming to develop food-packaging materials [2].

Depending on the source matrix, biobased food-packaging films can be separated into three main categories: polysaccharide-based, lipid-based, and protein-based, with polysaccharide-based films standing out, given their widespread availability in food wastes and by-products [3]. Examples include starch, cellulose, chitosan, galactomannans, and pectin, extracted from different wastes including fruit and vegetable peels and pomace [4,5,6,7], crayfish shells [8] and spent coffee grounds [9,10], among others. Although such biobased food-packaging films are renewable, biodegradable and biocompatible, and have a low carbon footprint, they can still present some characteristics that can prevent their use as food packages [2]. These include low hydrophobicity, mechanical strength, and thermal stability, as well as inadequate water and CO_2_ barrier properties, among others. To overcome some of the abovementioned issues, several studies have focused on the addition of nanofillers, biopolymers and oils that can also be waste based. These can act as reinforcing agents and plasticizers, improving mechanical strength, flexibility and barrier properties, or as additives for active packages, due to their antimicrobial, antioxidant and nutritional characteristics [2].

Vegetable oils are commonly incorporated into protein and polysaccharide-based biopolymer matrices to enhance properties such as hydrophobicity and water vapor barrier, as well as antimicrobial and antioxidant properties [11]. Lipids interact through physicochemical mechanisms such as hydrogen bonds and can alter the plasticity and crystallinity of the biopolymer films. In that regard, they can act as plasticizers and improve both the flexibility and processability of the material [12]. Also, their hydrophobic nature helps decrease the film solubility and the transmission of water vapor through the polymer [11]. Some recent examples of lipids added to edible films and coatings include soybean oil, coconut oil, pomegranate seed oil and linseed oil [13,14,15,16]. The prepared coatings and films were able to successfully increase the shelf life of several foodstuffs such as blueberries, cucumbers, strawberries and raw meat [11].

**Figure 1:**
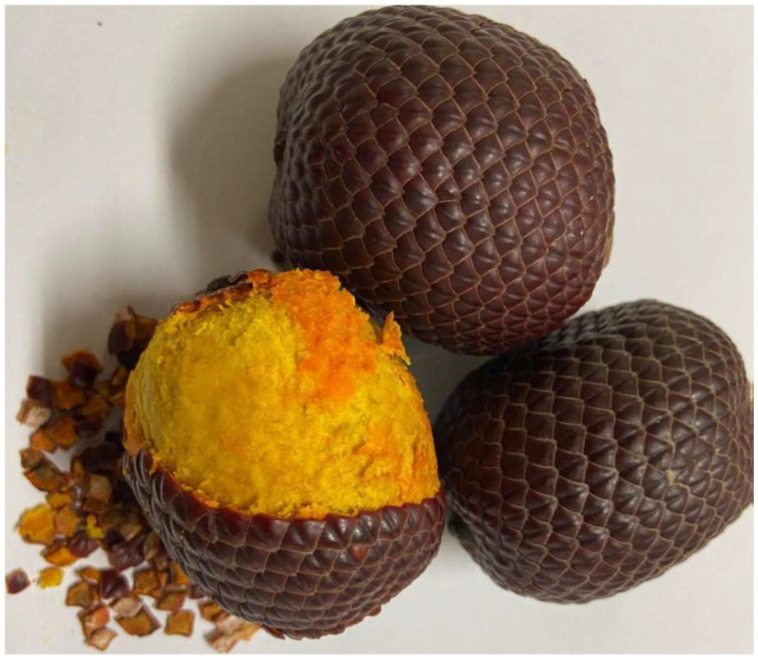
Buriti fruit.

Buriti (*Mauritia flexuosa* L.) is a fruit from the Brazilian Cerrado that presents a yellow-orange pulp (see Figure 1) of high nutritional value, with natural antioxidant compounds (phenolics, carotenoids and phytosterols), and high antioxidant capacity [17,18]. The oil extracted from the pulp (buriti oil) is rich in carotenoids, tocopherol and monounsaturated fatty acids, and has been extensively evaluated for applications in the pharmaceutical, cosmetics and food industries, with associated potential health benefits [19]. Furthermore, a few recent studies have evaluated buriti oil as an additive in polymeric materials [18,20].

Leite-Barbosa and collaborators [20] evaluated the potential of buriti oil (BO) as a plasticizer in linear low-density polyethylene (LLDPE) polymeric matrices. After molding, the samples were cooled down to 30 °C in a water-circulated press, maintaining the applied pressure of 10 tons. It was found that buriti oil acted as plasticizer and enhanced the fluidity of polymer chains. There was a small decrease in thermal stability, while mechanical properties (e.g., elongation at break and tensile strength) improved, especially for the sample with 0.5% BO. There was marked increase in surface hydrophobicity as the amount of added BO was increased. This study demonstrated the potential of buriti oil as an effective plasticizer for LLDPE polymeric matrices.

In a recent study by Assis et al. [19], BO was evaluated as an additive for starch-based films, with glycerol employed as plasticizer. Addition of BO led to a decrease in tensile strength and a significant increase in elongation at break. All the prepared films presented good thermal stability and rapid biodegradation (~15 days). Another study using commercially available cassava starch with added BO reported that increasing the amount of buriti oil led to decreases in water solubility, tensile strength, and elongation at break [21]. Results from these studies indicated that the effect of BO as a plasticizer will vary depending on the polymeric matrix.

Therefore, this work evaluated the use of buriti oil as a potential additive in biopolymeric films based on different carbohydrate-based matrices, namely galactomannans from locust bean gum (LBG) and starch (extracted from cassava peel and inner bark), both individually and blended. The films were prepared by casting and characterized in terms of their optical (color and opacity), barrier, and mechanical properties, as well as their biodegradability. This is the first study that evaluates the use of buriti oil in galactomannan-based films. Furthermore, our starch-based films employ agricultural waste as the carbohydrate source. Since BO could have a plasticizing effect or not depending on the polymeric matrix [20,21], we also evaluated its effect as a partial and total substitute for glycerol.

## 2. Materials and Methods

### 2.1. Materials

Cassava waste (periderm and cortex) was collected from a local market in Belo Horizonte, Brazil. The reagents employed for film preparation were locust bean gum, LBG (Sigma-Aldrich, St. Louis, MO, USA), glycerin P.A. 3-5-dinitrosalicylic acid (Synth, São Paulo, SP, Brazil), Arabic gum powder, density 1.35 (Êxodo Científica, Sumaré, SP, Brazil) and buriti oil, BO (lot MFR 24001) donated by Cooperativa Grande Sertão (Montes Claros, MG, Brazil).

### 2.2. Starch Extraction

Starch was extracted from the cassava peels using the methodology employed by Fronza and collaborators [22]. The peels were mixed with water (1:1 ratio) and ground in an industrial blender for 5 min. The resulting paste was subjected to additional homogenization for 30 min. This paste was subsequently filtered, and the filtrate was allowed to rest at 7 °C for 12 h. After resting, the supernatant was discarded, the decanted solids were washed multiple times and afterwards dried (24 h, 50 °C) and ground (D < 1.49 mm).

### 2.3. Film Preparation

The films were prepared by casting [6]. Locust bean gum (LBG) and cassava peel (periderm + cortex) starch were dissolved in distilled water under continuous stirring for 45 min at room temperature. Afterwards, glycerol was added, the mixture was heated up to 70 °C and kept under constant stirring for 1 h. Heating was then removed, and once the temperature was below 50 °C, the buriti oil emulsion was added. The BO emulsion was prepared by mixing the oil with Arabic gum and water using a probe ultrasonicator (Model QR500, Ultronique, Eco-Sonics, Indaiatuba, SP, Brazil ) operating at 20 KHz and 425 W for 3 min. The film solutions were placed in an ultrasonic bath (Model 1650 A, Unique-Ultra Cleaner, Indaiatuba, SP, Brazil-) for one hour and then transferred to square silicone molds. These were placed in a convective oven at 30 °C for 24 h, for solvent evaporation. The prepared films were then carefully removed from the molds and stored at room temperature [6]. Detailed information on the film’s composition is presented in Table 1. The LBG/starch ratio used in the combined film was chosen according to the study by Fronza and collaborators [6].

### 2.4. Film Characterization

#### 2.4.1. Chromatic Properties

Color parameters (L*, a*, b*) were measured using a tristimulus colorimeter (ColorFlex, Hunter Associates Laboratory, Reston, VA, USA) with standard D65 illumination and a normal colorimetric observer angle of 10°. Color intensity (c*, chroma) and tone (*h*, hue angle) were then calculated as:

(1) $$c^{*}={\left[{\left(a^{*}\right)}^{2}+{\left(b^{*}\right)}^{2}\right]}^{1/2}$$

(2) $$h={tan}^{-1}\left(b^{*}/a^{*}\right)$$

Opacity was calculated according to the method described by Alves et al. [23]. A small piece of film (4 × 1 cm) was placed on the inner wall of a quartz cuvette, and the absorbance was measured at 600 nm using a UV spectrophotometer (Biosystem, Curitiba, Brazil). The opacity was evaluated as:

(3) $$Opacity=A_{600}/X$$

where *A*_600_ is the absorbance value read at 600 nm and *X* is the film thickness, measured at 10 random points in each film sample, by a spheric face micrometer with 0.001 mm accuracy (Model 115-215, Mitutoyo Corporation, Kawasaki, Japan). Absorbance measurements were performed in triplicate, and an empty cuvette was used for establishing reference opacity value.

#### 2.4.2. FTIR Spectroscopy

Fourier Transform Infrared (FTIR) spectra of the film samples were obtained using a Thermo Scientific FTIR Spectrophotometer, Nicolet 6700 (Thermo Fisher Scientific, Waltham, MA, USA) with a DLATGS (Deuterated Triglycine Sulfate Doped with L-Alanine) detection and an Attenuated Total Reflectance (ATR) accessory with a ZnSe crystal. The scan range was 4000–600 cm^−1^ (16 scans per sample at 4 cm^−1^ resolution) and analyses were conducted in a controlled environment at 20 °C and 50% relative humidity.

#### 2.4.3. Thermal Profile

The thermal behavior of the films was evaluated by thermogravimetric analysis (TGA), based on the procedure described by Alves et al. [23]. TGA experiments were performed using TA Instruments Equipment (Model SDT Q600, New Castle, DE, USA). Samples (10 mg) were placed in aluminum pans and tests were carried out from 25 to 600 °C with an increasing rate of 10 °C min^−1^ under a nitrogen flow of 100 mL min^−1^.

#### 2.4.4. Mechanical Properties

Tensile strength (TS), elastic modulus (EM) and elongation at break (EB) were measured using a texture analyzer (TAXT Plus, Stable Micro Systems, Godalming, UK) employing a 5 kg load cell operating at a deformation speed of 0.8 mm per min. Film samples 10 mm × 100 mm in size were employed, and at least 5 replicates were performed for each film.

#### 2.4.5. Moisture Content (MC) and Water Solubility (WS)

Moisture content was evaluated according to a standard gravimetric method for which 2 cm × 2 cm film samples were dried at 105 °C for 24 h. Water solubility (*WS*) determination was based on the procedure described by Alves et al. [23]. Film samples were cut into circles (2 cm diameter), submitted to oven drying for 24 h at 105 °C and weighed afterwards (*W_i_*). A total of 30 mL distilled water was added, and the mixture was homogenized in an orbital shaker at 150 rpm for 24 h. Samples were dried and weighed again (*W_f_*). *WS* was calculated as:

(4) $$WS=\left(W_{i}-W_{f}/W_{i}\right)\times 100\%$$

#### 2.4.6. Barrier Properties

Water vapor transmission rate (*WVTR*) and water vapor permeability (*WVP*) were evaluated according to the methodology described by [24]. Glass vials containing previously dried silica (~1/3 vial volume) were covered with the prepared films and placed in a controlled environment (20 ± 2 °C at 75% relative humidity). The vials were weighed every 24 h during seven days. The variation in silica weight gain with time was plotted and *WVTR* and *WVP* (g/s·m·Pa) were calculated as follows:

(5) $$WVTR=\frac{w}{t}\times \frac{1}{A}$$

(6) $$WVP=WTR\times \frac{\delta }{∆P}$$

where *w*/*t* is the slope of the weight gain line as a function of time (g/day); *A* is the sample permeation area (m^2^); *δ* corresponds to the sample thickness (mm); and Δ*P* is the water vapor saturation pressure at the experiment temperature (2.33921 kPa).

Oxygen permeability (*OXP*) determination was based on the methodology described by Cheng and collaborators [25]. Glass vials (15 mL) containing 3 g of deoxidizer reactant (iron powder, activated carbon, and sodium chloride) were covered with the prepared films and placed in a desiccator containing a saturated solution of barium chloride (99% RH) for 48 h at 25 °C. The initial (*W_i_*) and final (*W_f_*) weights were recorded, and OXP was evaluated as follows:

(7) $$OXP=\left(W_{f}-W_{i}\right)/\left(t\times A\right)$$

where *t* is the equilibration time (h), and *A* is the film area (m^2^).

#### 2.4.7. Film Biodegradation

Biodegradability tests were based on the methodology described by Fronza et al. [6] with soil preparation following the guidelines of ASTM G160-03 [25]. The film samples (2 × 2 cm) were buried in soil previously placed in plastic containers that were maintained at ambient temperature. Water was sprayed regularly to maintain soil humidity and film samples were removed and analyzed daily, until complete film degradation.

#### 2.4.8. Statistical Analysis

Experiments were performed in triplicate, with at least 3 measurements per sample, and Minitab 19.2020.1.0 software was employed for analysis of variance (ANOVA) and Tukey analysis [23].

## 3. Results and Discussion

### 3.1. Chromatic Properties

**Figure 2:**
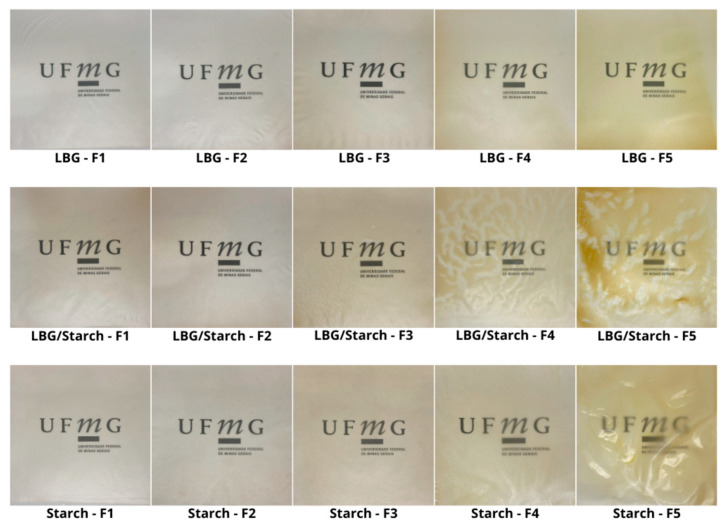
Photographic images of the prepared films. F1 = control film; F2 = control film with added emulsifier; F3 = film with BO as additive; F4 = film with BO as additive/plasticizer (partial glycerol replacement); F5 = film with BO as plasticizer (total glycerol replacement).

The prepared films can be viewed in Figure 2 and the corresponding color parameters are displayed in Table 2. Film appearance varies depending on the type of polysaccharide and on the amount of added emulsified buriti oil. Luminosity values, that can range from 0 (black) to 100 (white), are low, varying from 26.5 to 33.3. There was a slight increase in luminosity for films containing LBG with the addition of buriti oil, thus suggesting that oil incorporation led to better light dispersion, leading to films that were lighter and more transparent (notice the corresponding decrease in opacity). However, the increase in luminosity with increasing BO concentration was not observed for films based on pure starch, probably in association with the increase in film thickness, as also reported by a previous study [19]. In general, addition of vegetable oils tends to increase the thickness of biopolymer-based packaging materials. Such increase in film thickness could be associated with conformational changes in the polymeric matrix resulting from the incorporation of BO. Nonetheless, an increase in oil concentration will not necessarily result in an increase in film thickness, as observed for the galactomannan films. As previously reported in the literature, the effect of adding a vegetable oil will vary depending on the composition of the film matrix [11].

The color tone, represented by the hue angle, tends to yellow (~90°), with a slight increase with the addition of BO. Visually, this yellow tone is more perceptible for films with added BO, due to the increase in color saturation (c*). This increase in color saturation and the corresponding yellow tone is attributed to BO being rich in carotenoids [17]. Notice, however, that for starch-based films with added BO, a degree of heterogeneity is observed when BO is used as a replacement for glycerol (F4, F5). This was not observed for the galactomannan-based films, that showed good dispersion of the emulsified BO droplets within the film-forming matrix, regardless of the plasticizer. Thus, the addition of BO will affect color parameters because of interactions between oil droplets and the film-forming matrix [26]. It should be noted that, because of differences in the fatty acid profiles, different plant oils, including buriti, present different degrees of hydrophobicity [11]. Similarly, the hydrophilic nature of polysaccharides will vary depending on the available polar groups, such as hydroxyl, amino, and carboxyl, among others. This will have a direct impact on the ability of BO to disperse uniformly within the biopolymer matrices.

### 3.2. FTIR Spectroscopy

To properly analyze the spectra for the films prepared in this study, it must be understood beforehand that the thermal (70 °C for 1 h) and ultrasonic (25 kHz and 135 W) treatments to which the filmogenic solutions were subjected to promoted a certain degree of starch gelatinization, allowing for a low degree of crystallinity in the solution. Both heat and ultrasound treatments are widely known to decrease the extent of crystalline structures in starch [27].

**Figure 3:**
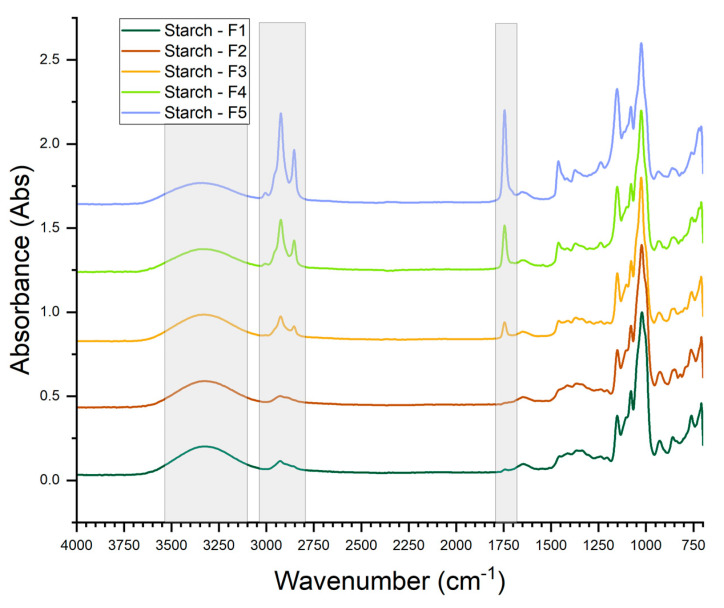
FTIR spectra for the pure-starch-based films.

The normalized FTIR spectra for the pure-starch-based films are presented in Figure 3. FTIR is considered to be sensitive solely to the starch short-range order structures [28], defined as the double helical order; thus, changes in the spectra can only reflect the interactions of starch and added components at the short-range ordered structures. All the spectra exhibit a broad band in the range of 3600–3100 cm^−1^ associated with the O–H stretching vibrations of hydrogen-bonded polysaccharides. No significant changes were observed in this range for the distinct film formulations. There was a slight blue shift in the band maximum as the content of Arabic-gum-emulsified buriti oil was increased in the film’s formulations. This blue shift is herein attributed to the probable hydrogen-bond interactions between the Arabic gum and the double helix structures of the starch molecules. The bands in the range 2980 to 2850 cm^−1^ are attributed to asymmetric and symmetric stretching vibrations of –CH_2_ groups. There is a clear increase in the intensity of these bands as the oil content is augmented, related to the increased presence of saturated hydrocarbon chains in the oil molecules, such as palmitic acid. The appearance and intensity increase in a band at ~1750 cm^−1^ as the oil content was increased is attributed to carbonyl (–C=O) stretching vibrations in triglyceride esters. It may also be associated with the phenolic acids present in buriti oil [29]. The slightly broad band at ~1650 cm^−1^ is attributed to the H–O–H bending vibration of the tightly absorbed water in the starch structures [30]. A noticeable band at 1438 cm^−1^ appear in the spectra of the films with incorporated emulsified buriti oil with increased intensity as the oil content was increased in the film formulation. This band can be assigned to symmetrical stretching of carboxylic groups of uronic acid residues in Arabic gum or to C–H scissoring and bending in saturated hydrocarbons, such as palmitic acid in the triglyceride fraction of the buriti oil [29]. A band appeared at 1237 cm^−1^ when the emulsified oil was added to the film formulation and its intensity increased as the oil content was increased. This band can be assigned either to the vibrations of saturated esters C–C(=O)–O or to the asymmetric stretching of the P=O bond in phospholipids.

In the fingerprint region of the spectra, i.e., 1200–800 cm^−1^, the bands were significantly overlapped, and the second derivatives of the spectra were taken to improve resolution of the bands. Also, the second derivative of FTIR spectra improves the sensitivity to chemical changes in the sample analyzed [31]. In the second derivative of the spectra, the bands were well-resolved and symmetrical, allowing for the visualization of several distinct bands that are characteristic of starches and of the added Arabic-gum-emulsified buriti oil. A well-resolved band at ~1152 cm^−1^ was assigned to the stretching vibrations of C–O bonds in C–OH groups in the glucose rings of starch. However, as the oil content increased in the film, this band broadened and became a bit skewed towards a slightly higher wavenumber, possibly due to the contribution of molecular arrangements similar to those of triolein present in the triglyceride fraction of the buriti oil [29]. A band at 1119 cm^−1^ appeared in the spectra second derivative when the oil was introduced in the film formulation. As the oil content increased this band’s intensity also increased, which can be regarded as the contribution of C–O stretching vibrations of ester groups within the triacylglycerol molecules in the oil. In all the spectra for the pure-starch-based films, there are three well-resolved bands at 1080–1079, at 1057–1048, and at 1025–1022 cm^−1^. The bands at 1080–1079 and 1057–1048 cm^−1^ were assigned to the C–O bending vibration within the anhydro glucose ring structure of starch and, together with the bands at 1025–1022 cm^−1^, can be closely associated with the short-range order of the starch structures (i.e., the local organization of helices into crystalline arrays) [31].

The absorbance band at 1057–1048 cm^−1^ is sensitive to the short-range-order crystalline structure of starch (i.e., double helix), whereas the band at 1025–1022 cm^−1^ is sensitive to the amorphous regions in the starch. The ratio of absorbance intensities of the bands at 1048 and 1022 cm^−1^, $R_{1048/1022}$, is commonly used in the literature to evaluate the changes in the short-range structures of starch [28]. The higher the ratio $R_{1048/1022}$, the higher the short-range crystallinity of starch [31]. $R_{1048/1022}$ values were herein calculated for the pure-starch-based films and are presented in Table S1 (Supplementary Material). With the addition of Arabic gum to the starch filmogenic solution, the ratio significantly decreased, demonstrating that Arabic gum hindered the recrystallization (retrogradation) of starch, reflecting a strong interaction between the gum and the starch molecules. With the addition of the emulsified oil, the ratio $R_{1048/1022}$ decreased slightly in comparison with that of the pure-starch film, demonstrating that a partial recrystallization of starch had occurred. No significant differences in the absorbance intensities ratio were observed when the emulsified oil content (and consequently the Arabic gum content) was increased in the film, indicating a higher affinity of the Arabic gum for the oil than for the starch molecules.

Well-defined bands can be viewed in the 995–998 cm^−1^ range and are assigned to the intramolecular hydrogen bonding of the O–H group at C6 of the glucose ring in starch. A group of bands appear in the 866–855 cm^−1^ range and are characteristic wavenumbers of α- anomeric carbon configurations in starch.

**Figure 4:**
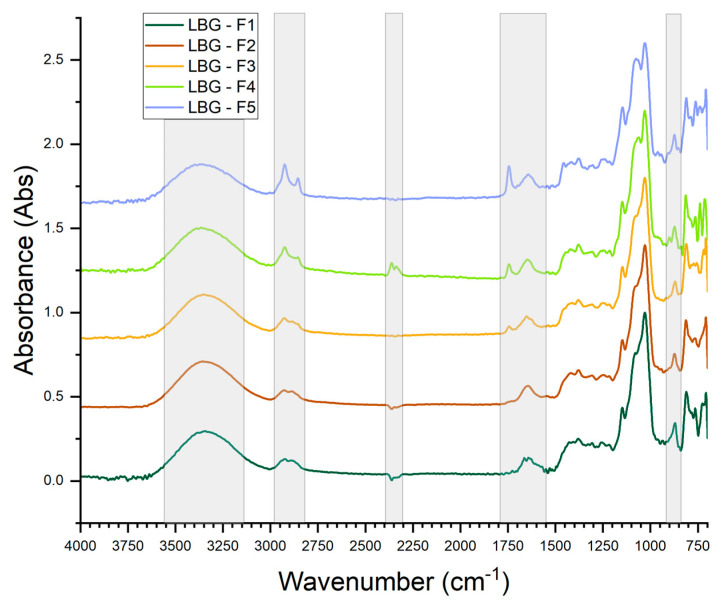
FTIR spectra for the pure-LBG-based films.

The normalized FTIR spectra for the pure-LBG-based films are presented in Figure 4. Like the spectra for the pure-starch-based films, all the spectra for pure-LBG-based films exhibited a broad band in the range of 3600–3100 cm^−1^ associated with the O–H stretching vibrations of galactomannan and water. No significant changes were observed in this range for the distinct film formulations. Again, there was a slight blue shift in the band maximum as the content of Arabic-gum-emulsified buriti oil was increased in the films’ formulations. This blue shift is herein attributed to the hydrogen-bond interactions between Arabic gum and galactomannan molecules. The bands in the range 2980 to 2850 cm^−1^ are attributed to asymmetric and symmetric stretching vibrations of –CH_2_ groups, with an increase in the bands’ intensities as the oil content was increased being attributed to the increased content of saturated hydrocarbon chains in the oil molecules, such as palmitic acid.

The introduction of the emulsified buriti oil into the film formulations led to the appearance of a sharp band at ~1750 cm^−1^ associated with the carbonyl (–C=O) stretching vibrations in triglyceride esters and its intensity increased with the increase in oil content in the film. There is a broad peak at ~1640 cm^−1^ associated with the H–O–H bending vibration of the absorbed water in the galactomannan and Arabic gum structures. The range 1400–700 cm^−1^ comprises a cluster of overlapping bands encompassing the fingerprint regions of the spectra. Again, the second derivatives of the spectra were taken to allow for a better resolution of the bands. The band at ~1370 cm^−1^ was assigned to C–H vibrations and CH2 bending in galactomannans and cellulose. A band at 1149–1151 cm^−1^ was assigned to O–C–O stretching vibrations of mannose-containing polysaccharides [30]. This band decreased in intensity as the content of emulsified oil was increased in film formulation. A cluster of bands appears at 1024–1033 cm^−1^ and are assigned to C–O stretching and C–C stretching of C6–H2–O6 in cellulose and galactomannan. The presence of a small amount of cellulose is expected given the inherent difficulty in isolation and purification of galactomannans from cellulosic materials. With the addition of emulsified oil in Arabic gum, a band at 1039 cm^−1^ appears and is assigned to C–C stretching in galactose rings of galactans (i.e., Arabic gum) [30].

Bands in the immediate vicinity of 900 cm^−1^ are assigned to β-configuration of the anomeric carbon in the mannose and galactose rings and bands at 877–871 cm^−1^ are attributed to C1–H bending in galactomannan. The bands below 800 cm^−1^ are attributed to the diverse modes of skeletal vibrations of polysaccharides [30].

**Figure 5:**
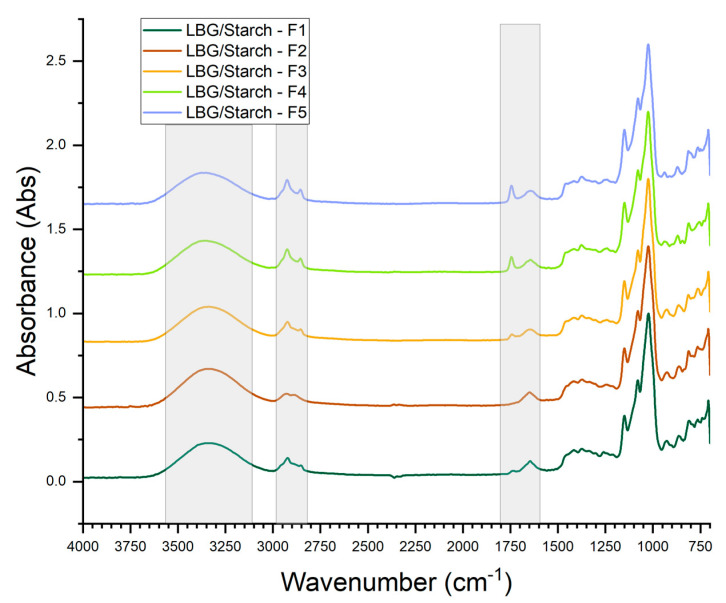
FTIR spectra for the LBG/starch-based films.

The normalized FTIR spectra for the LBG/starch-based films are presented in Figure 5. The second derivatives of the spectra were taken to improve the resolution of bands and to improve the sensitivity to chemical interactions [31]. Most of the bands in the spectra are common to either the spectra for pure-starch-based or the spectra for pure-LBG-based films. The bands at 1055 and 1022 cm^−1^ are of particular interest since they reflect the effects of mixing starch, LBG and emulsified oil on the retrogradation of starch during film formation. $R_{1048/1022}$ were calculated for the LBG/starch-based films and are presented in Table S1 (Supplementary Material). The ratio $R_{1048/1022}$ for the LBG/starch F1 formulation was significantly smaller than that for the pure-starch F1 formulation, indicating strong interactions between LBG and starch molecules that hinder retrogradation of starch during film formation. The addition of Arabic gum to the LBG/starch-based film formulation promoted a further decrease in $R_{1048/1022}$ value, indicating that Arabic gum molecules also hindered retrogradation of the starch during film formation. The addition of Arabic-gum-emulsified oil and a further increase in its content in the film formulation (F3, F4 and F5) allowed for a higher degree of retrogradation in comparison to those for LBG/starch F1 and F2 formulations but with values of $R_{1048/1022}$ significantly lower than those for the starch-based formulations. Again, Arabic gum was demonstrated to have higher affinity to the buriti oil or to the galactomannans than to the starch molecules.

To properly analyze the FTIR spectra of the prepared films, it is important to call attention to the fact that, aside from the base polysaccharides, i.e., starch, galactomannan and glycerol, the films also comprise emulsified oil droplets, with Arabic gum being the emulsifying agent. Arabic gum comprises a polysaccharide moiety with a 2% proteinaceous moiety attached to it. The polysaccharide fraction comprises β-(1 → 3)-D-galactopyranosyl units branched at the O6 position by side chains composed of β-D-galactopyranosyl and α-L-arabinofuranosyl units. The proteinaceous fraction comprises arabinogalactan polipeptides and proteins, and glycosylated proteins. These in turn mostly comprise hydroxyproline, serine, proline and threonine residues [32]. The hydrophobic interactions in the proteinaceous fraction of Arabic gum are deemed the major driving force for the emulsifying ability of Arabic gum [33]. Therefore, a thorough inspection of the amide-I (1700–1600 cm^−1^) and amide-II (1600–1500 cm^−1^) regions is needed, to identify and analyze the secondary structures of the polypeptide and protein fraction.

The absorbance band at ~2980 cm^−1^ that becomes more prominent as the emulsified oil content is increased in the film formulation could be assigned to the stretching vibration of COO–H and –NH_3_+ groups in the amide-B region [23]. The amide-I region of the spectra, i.e., 1700–1600 cm^−1^, is highly sensitive to the conformational state of proteins, with the individual bands in this region strictly correlating to distinct secondary structures of the polypeptide chains [34]. However, an inspection of such regions in all the spectra reveals a significant overlapping of bands and their second derivatives (not shown) were assessed to improve resolution and allow for the identification of individual bands.

A significant number of peaks were revealed in the second derivatives of all spectra, throughout the entire amide-I and amide-II regions, indicating the presence of all known secondary structures of proteins, including polyproline helices (PPII) associated with bands in the region 1625–1615 cm^−1^ [33]. The bands in the second derivative spectra are reasonably symmetrical. Therefore, a qualitative analysis of their behavior, based solely on band heights, is sufficient to provide a notion of the types of changes occurring in the protein moiety as the content of emulsified oil is increased in the film.

In the second derivative of the FTIR spectrum for the film comprised solely of LBG (F1), the number of bands in the amide-I and amide-II regions is insignificant, for there was a negligible amount of proteinaceous material in the matrix polysaccharide used. In the spectra for the films in which locust bean galactomannan is the base polysaccharide and emulsified oil is included, the bands at 1549–1545 cm^−1^ (amide-II region) in conjunction with the bands 1660–1650 cm^−1^ (amide-I region) are tentatively assigned to α-helix structures and the bands’ heights increased as the emulsified oil content increased. The bands at 1625–1615 cm^−1^ (amide-I region) are herein assigned to either PPII helices or aggregation prone β-sheets (low frequency) [34], and their heights increased as the emulsified oil content increased. The same behavior was observed for β-turns associated with the bands at 1690–1680 cm^−1^ and for the aggregation-prone β-sheets (high frequency) at 1700–1690 cm^−1^. However, differently from the behavior of α-helix associated bands, the increase in β-sheets bands heights was not proportional to the increase in emulsified oil content in the film formulation, with the associated bands in the amide-II region (1535–1530 cm^−1^) presenting an erratic behavior. Since erratic behavior was also observed for the bands associated with unordered structures (or random coils), 1648–1639 cm^−1^, one can speculate that, due to their less stable nature, a fraction of the β-sheets might have transitioned to unordered structures during the filmogenic solution preparation and the film-forming step of the procedure. Aside from the bands associated with the secondary structures of the proteinaceous fraction of the films, a significant number of bands were observed in the second derivative spectra that are herein tentatively attributed to the absorbance of particular amino acids and their respective side chains [35].

In the spectra for starch-based films, the number of bands in the amide-I and amide-II regions is significantly higher than in the same region of the spectra for galactomannan-based films. This can be explained by the fact that the matrix starch used for the film formulation already contained a proteinaceous fraction, that might have originated from the cassava waste extraction process, and for which the protein secondary structures have their absorbance wavenumber maxima shifted in relation to those of the proteinaceous fraction of the Arabic gum due to distinct amino acid composition and distinct interactions between molecules. All the secondary-structure-associated bands are present in the second derivative of the spectra for the starch-based films; however, the behavior of the individual bands does not present a direct correlation with the changes in film formulation regarding the addition of emulsified buriti oil, i.e., they do not necessarily increase in height with the increase in emulsified oil content. Hence, a decent analysis of the starch-based film spectra in this region is not feasible without the support of other analytical techniques, such as nuclear magnetic resonance and X-ray diffraction, which were not available for this work.

In the spectra for LBG/starch-based films, the number of bands in the amide-I and amide-II regions is smaller than in the same region of the spectra for starch-based films but greater than in the same region of the spectra for galactomannan-based films. Again, all the bands associated with the secondary structures are present; however, with a non-correlating behavior regarding the content of emulsified oil in the film formulation. Given the compositions of the films and the conditions applied during their preparations (e.g., heat and ultrasonic treatments), a multitude of interactions and structure rearrangements of their components are expected to occur, making it difficult to visualize the intricate transitions of specific secondary structures into others.

### 3.3. Thermal Profile

**Figure 6:**
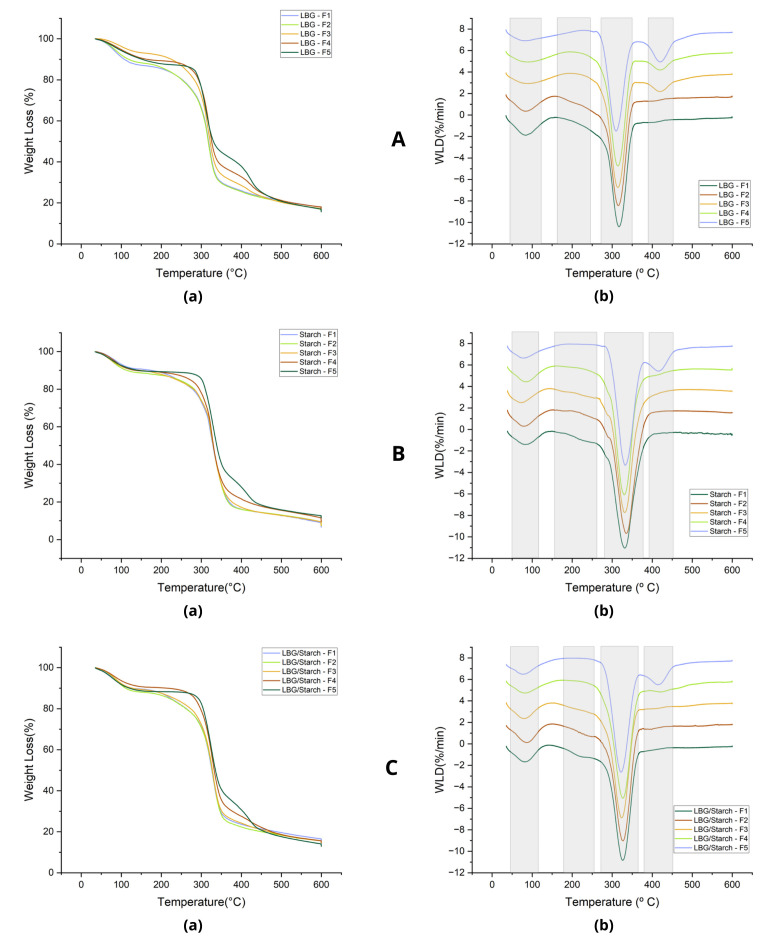
(**a**) Weight loss curves and (**b**) their derivatives for (**A**) LBG, (**B**) starch and (**C**) LBG/starch films. F1 = control film; F2 = control film with added emulsifier; F3 = film with BO as additive; F4 = film with BO as additive/plasticizer (partial glycerol replacement); F5 = film with BO as plasticizer (total glycerol replacement).

The thermal behavior of the prepared films can be viewed in Figure 6, specifically the variation in weight loss with temperature (Figure 6a) and the corresponding weight loss derivative curves (Figure 6b). The curves presented in Figure 6 show two or three significant thermal events, depending on the film. The first decomposition stage, in the range of 71 to 94 °C, is attributed to water evaporation, and is present in all the prepared films. The corresponding weight losses are in the range of 7 to 13%. A small thermal event in the range of 198 to 254 °C can be observed in all films except F5, being associated with glycerol decomposition [36]. The main decomposition event occurred in the following temperature ranges: 310 to 317 °C for LBG films; 328 to 331 °C for starch films; and 325 to 327 °C for LBG/starch films. The small variation in degradation temperature range for each type of film indicates that the addition of BO did not have a significant effect on film stability. Another thermal event took place in the range of 413 to 423 °C, in association with the decomposition of buriti oil. It was more pronounced in the samples where glycerol was replaced by BO (F5).

### 3.4. Mechanical Properties

**Figure 7:**
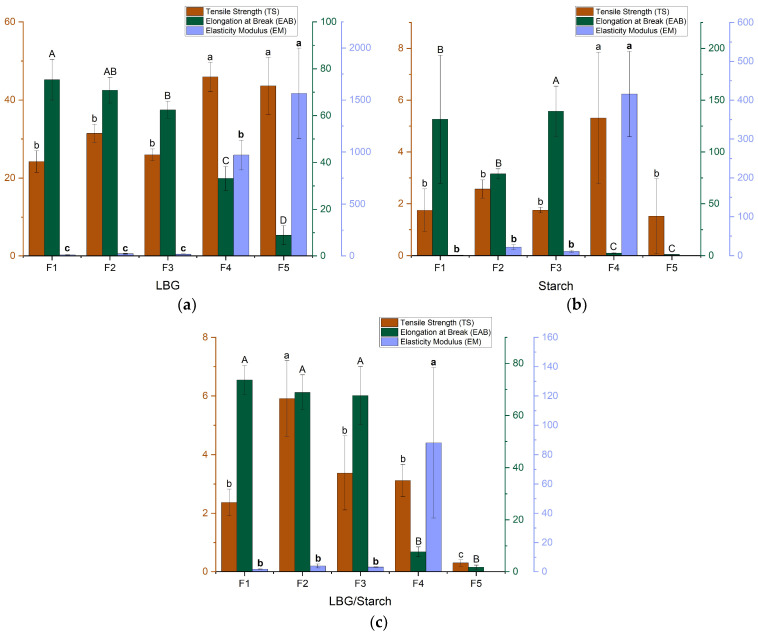
Mechanical properties of the prepared films: (**a**) LBG; (**b**) starch; (**c**) LBG/starch. F1 = Control film; F2 = control film with added emulsifier; F3 = film with BO as additive; F4 = film with BO as additive/plasticizer (partial glycerol replacement); F5 = film with BO as plasticizer (total glycerol replacement). Different letters indicate significant differences for each mechanical property.

The mechanical properties of the prepared films are displayed in Figure 7. It can be observed that these properties varied significantly depending on the base polymer and on the added amount of buriti oil. For the galactomannan-based film (Figure 7a), incorporation of BO while maintaining glycerol as plasticizer (F3) did not have a significant effect on tensile strength or elasticity modulus but resulted in a small reduction in elongation at break. When BO was used as a glycerol substitute (F4, F5), both the tensile strength and elasticity modulus increased with the substitution percentage, but there was a considerable decrease in elongation at break. The same behavior was reported when glycerol was reduced in another study using LBG films [37]. The decrease in glycerol increased the interaction between the polymeric matrix constituents, resulting in a more compact and stiffer film. The fact that BO did not counteract this effect indicates that it did not work as a plasticizer in a galactomannan polymeric matrix. In the starch-based film (Figure 7b), adding BO without removing glycerol led to a slight increase in elongation at break without a significant effect on the other properties. Partial substitution of glycerol (F4) led to a significant increase in elasticity modulus and a drastic decrease in elongation at break. However, the elasticity modulus also decreased significantly when glycerol was totally replaced. The same behavior was observed for the combined starch–galactomannan films (Figure 7c). This indicates that BO did not act as a plasticizer and was poorly dispersed in the polymer matrix, in accordance with film characteristics displayed in Figure 2. Notice that, regardless of the use of BO, the films containing starch present much lower mechanical resistance in comparison with the films based on LBG. Nonetheless, tensile strength, elasticity modulus and elongation values obtained for the control films are consistent with those previously reported in the literature [38]. It should be pointed out that the type of oil being employed will also cause variations in mechanical properties. For instance, for starch-based films, adding sunflower oil decreased TS, while adding coffee oil had the opposite effect [39,40].

The inability of the emulsified oil to act as a plasticizer can be explained as follows. Arabic gum was used as emulsifier for buriti oil. Arabic gum comprises a polysaccharide backbone with polypeptide/protein attached to it. The polypeptide/protein fraction in turn comprises three distinct fractions: (i) arabinogalactan-peptide; (ii) arabinogalactan-protein; and (iii) glycoprotein. The arabinogalactan-protein fraction is deemed responsible for the emulsifying and interfacial properties of the gum [33]. Hydroxyproline is the most abundant amino acid in the protein fraction. An analysis of the protein moiety of the gum revealed the presence of aggregations caused by hydrophobic CH…π interactions between the hydroxyproline (and proline) imino acid and the aromatic amino acids tyrosine and phenylalanine of the protein chain. The amino acids involved in the CH…π hydrophobic interactions are attracted to the hydrophobic oil phase, and the hydrophilic polysaccharide fraction of the molecules arrange themselves towards the water phase, the mechanism in which the emulsion is stabilized [33]. Arabic gum can be adsorbed on the oil droplets in multilayers, creating a thick involucre around the droplet, hindering possible direct interactions of the oil with any moiety outside the polysaccharide outer shell. On the other hand, the polysaccharide moiety in the outer shell of the emulsified droplet can establish hydrogen-bonding interactions with the film matrix polysaccharides (i.e., starch and galactomannan) in a way that an overall plasticizing action is precluded and the film becomes stiffer; hence, the observed increase in tensile strength and elasticity modulus. If the oil droplets were not obstructed from interacting with the matrix polysaccharide moiety, possible lipid–starch complexes could be formed due to interactions between the lipids and the hydrophobic helical cavities in the amylose structure [41], and the film could be stiffer than it currently is.

### 3.5. Moisture Content (MC), Water Solubility (WS) and Barrier Properties

Moisture content and water solubility are key parameters for evaluation of film stability in aqueous systems, and these parameters are shown in Table 3. The highest moisture and solubility values were observed for the control films, with a significant reduction with the addition of buriti oil. This behavior was expected given the opposite polarity of glycerol (hydrophilic) and buriti oil (hydrophobic). As a plasticizer, glycerol establishes hydrogen bonds with the polymeric chains. As the amount of glycerol diminishes, the diffusion of the plasticizer decreases and the access of water molecules to polar groups in the polymeric matrix is minimized. There is a strong interaction between polymeric chains, increasing film hydrophobicity and its rigidity, as observed in the results of the mechanical properties [37]. Galactomannan-based films presented higher water solubility than the starch-based ones, because starch is not as hydrophilic as LBG.

Water barrier properties, namely water vapor transmission rate (WVTR) and permeability (WVP), were higher for the LBG control film in comparison to the ones that contained starch. Nonetheless, the addition of BO provided a significant improvement in these barrier properties for the LBG films, whereas there was no significant variation for the films containing starch. The reduction in WVP and WVTR after the introduction of BO can be attributed to the increase in film hydrophobicity and in the tortuosity of the polymeric matrix that hinders the transport of water molecules [42]. Oxygen is an important environmental factor that can cause spoilage and deterioration of food during storage. This could be related to the oil composition, given that adding either sunflower or coffee oil led to starch-based films demonstrating WVP values [39,40]. Oxygen permeability (OXP) decreased significantly with the addition of buriti oil, indicating that BO also acts as an oxygen barrier. In the case of pure starch films, BO did not have a significant effect on water barrier properties. Oxygen permeability, on the other hand, decreased significantly with the addition of the emulsifier and buriti oil. The compatibility of Arabic gum and buriti oil with the film matrix resulted in a dense structure, thus inhibiting the transport of non-polar oxygen molecules through the film [43]. Previous studies using films based on cassava starch have reported decreases in WVP with the addition of oils, given the associated hydrophobicity [44]. Barrier properties did not vary significantly with BO addition for the combined starch–galactomannan films, although moisture and solubility values were significantly reduced.

### 3.6. Film Biodegradation

**Figure 8:**
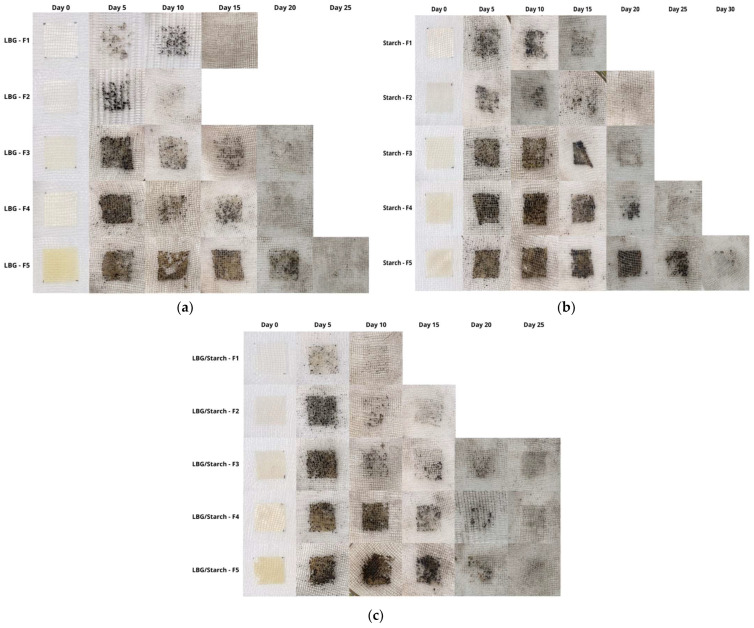
Biodegradability results for the prepared films. (**a**) LBG; (**b**) starch; (**c**) LBG/starch. F1 = control film; F2 = control film with added emulsifier; F3 = film with BO as additive; F4 = film with BO as additive/plasticizer (partial glycerol replacement); F5 = film with BO as plasticizer (total glycerol replacement).

Film biodegradability was accessed through an evaluation of the changes in film appearance with time, as they remained in contact with moist soil (see Figure 8). Mass loss with time was not measured, given that soil particles remained attached to the samples. Attempts to remove the adhered soil particles damaged the films, with consequent sample mass losses and underestimation of film weight. However, it can be seen from Figure 8 that all the prepared films degraded completely after 30 days. The control films (F1) degraded faster, with degradation times ranging from 10 to 15 days. Other studies using LBG and starch-based films report a wide range of degradation, from 3 to 30 days [23,45], with shorter times reported for LBG. The control LBG/starch conjugated film degraded faster than the pure ones. It seems that, in the blend, LBG degraded first, exposing starch molecules and favoring degradation. Films containing BO, either as an additive or a replacement for glycerol, took more time to degrade. This is attributed to the increase in hydrophobicity associated with adding oil to the film and the consequent decrease in water absorption and microbial activity. These results are consistent with water solubility and moisture content data.

## 4. Conclusions

Biodegradable films were produced using starch, galactomannan, and buriti oil obtained from cassava, locust bean, and buriti by-products. All films showed low opacity and a yellowish color, with buriti oil decreasing opacity but increasing color intensity. FTIR analysis revealed that galactomannan significantly reduced starch retrogradation, lowering crystallinity, and that emulsified buriti oil further hindered retrogradation in both starch-only and starch–galactomannan films. Buriti oil had no major impact on thermal stability. Increasing the amount of emulsified oil enhanced tensile strength and stiffness but reduced elongation at break, due to interactions of the Arabic gum polysaccharide moiety with the matrix polysaccharides. Starch-based films were mechanically weaker than those made only with galactomannan. Buriti oil enhanced water resistance and barrier properties, particularly in galactomannan films. All films were biodegradable, though the presence of buriti oil slowed the degradation process.

## Figures and Tables

**Table 1:** Mass composition of the prepared films.

Carbohydrate	Film	LBG	Starch	Glycerin	Buriti Oil	Arabic Gum
	F1	3 g	-	0.6 g	-	-
	F2	3 g	-	0.6 g	-	0.045 g
LBG	F3	3 g	-	0.6 g	0.15 g	0.045 g
(galactomannan)	F4	3 g	-	0.3 g	0.3 g	0.09 g
	F5	3 g	-	-	0.6 g	0.18 g
	F1	-	3 g	0.6 g	-	-
Starch	F2	-	3 g	0.6 g	-	0.045 g
	F3	-	3 g	0.6 g	0.15 g	0.045 g
	F4	-	3 g	0.3 g	0.3 g	0.09 g
	F5	-	3 g	-	0.6 g	0.18 g
	F1	0.75 g	2.25 g	0.6 g	-	-
LBG/Starch	F2	0.75 g	2.25 g	0.6 g	-	0.045 g
(25:75)	F3	0.75 g	2.25 g	0.6 g	0.15 g	0.045 g
	F4	0.75 g	2.25 g	0.3 g	0.3 g	0.09 g
	F5	0.75 g	2.25 g	-	0.6 g	0.18 g

F1 = control film; F2 = control film with added emulsifier (Arabic gum); F3 = film with BO as additive; F4 = film with BO as additive/plasticizer (partial glycerol replacement); F5 = film with BO as plasticizer (total glycerol replacement).

**Table 2:** Chromatic parameters of the prepared films.

					Opacity	Thickness
Carbohydrate	Film	L*	c*	h*	(mm^−1^)	(mm)
	F1	28.22 ± 0.82 ^c^	5.27 ± 0.19 ^cd^	90.35 ± 0.84 ^a^	13.920 ± 0.136 ^a^	0.046 ± 0.006 ^b^
LBG	F2	27.34 ± 0.34 ^c^	4.61 ± 0.26 ^d^	86.99 ± 1.76 ^b^	11.702 ± 0.526 ^b^	0.056 ± 0.006 ^a^
(galactomannan)	F3	30.49 ± 0.49 ^b^	6.03 ± 0.21 ^bc^	90.93 ± 1.03 ^a^	11.236 ± 0.188 ^b^	0.056 ± 0.006 ^a^
	F4	31.10 ± 1.29 ^b^	6.50 ± 0.52 ^b^	91.46 ± 0.55 ^a^	11.585 ± 0.569 ^b^	0.056 ± 0.006 ^a^
	F5	33.34 ± 0.35 ^a^	9.60 ± 0.556 ^a^	90.57 ± 0.35 ^a^	14.146 ± 1.235 ^a^	0.057 ± 0.009 ^a^
	F1	20.91 ± 1.45 ^c^	8.63 ± 0.08 ^a^	82.57 ± 1.83 ^b^	21.461 ± 1.137 ^a^	0.038 ± 0.007 ^c^
Starch	F2	30.49 ± 0.83 ^a^	6.18 ± 0.20 ^d^	82.55 ± 0.96 ^b^	11.906 ± 1.211 ^c^	0.039 ± 0.005 ^c^
	F3	27.45 ± 0.16 ^b^	6.22 ± 0.12 ^d^	85.63 ± 1.58 ^ab^	12.765 ± 1.503 ^bc^	0.048 ± 0.004 ^b^
	F4	26.46 ± 0.54 ^b^	6.90 ± 0.18 ^c^	88.26 ± 1.57 ^a^	15.591 ± 0.574 ^b^	0.048 ± 0.005 ^b^
	F5	26.69 ± 1.81 ^b^	7.77 ± 0.22 ^b^	88.09 ± 0.85 ^a^	10.720 ± 1.089 ^c^	0.058 ± 0.008 ^a^
	F1	24.86 ± 1.53 ^c^	5.04 ± 0.85 ^b^	86.50 ± 1.50 ^a^	16.422 ± 0.503 ^a^	0.041 ± 0.014 ^b^
LBG/Starch	F2	27.21 ± 0.33 ^bc^	5.07 ± 0.22 ^b^	85.84 ± 3.19 ^a^	16.266 ± 0.230 ^b^	0.042 ± 0.006 ^b^
(25:75)	F3	30.71 ± 0.70 ^a^	10.02 ± 0.12 ^ab^	86.41 ± 0.27 ^a^	16.505 ± 0.237 ^a^	0.046 ± 0.004 ^b^
	F4	29.47 ± 0.34 ^ab^	7.13 ± 0.76 ^ab^	87.73 ± 0.47 ^a^	9.670 ± 0.265 ^c^	0.076 ± 0.015 ^ab^
	F5	31.86 ± 2.16 ^a^	14.29 ± 6.82 ^a^	87.25 ± 3.23 ^a^	9.679 ± 0.115 ^c^	0.088 ± 0.055 ^a^

F1 = control film; F2 = control film with added emulsifier; F3 = film with BO as additive; F4 = film with BO as additive/plasticizer (partial glycerol replacement); F5 = film with BO as plasticizer (total glycerol replacement). Means with the same letters in the same column for a specific basis carbohydrate do not show statistical differences (*p* > 0.05).

**Table 3:** Moisture content and solubility in water and film barrier properties.

Carbohydrate	Film	MC (%)	WS (%)	WVTR × 10^3^ (g/s·m^2^)	WVP × 10^10^ (g/s·m·Pa)	OXP × 10^9^ (g/s·mm^2^)
	F1	11.748 ± 0.307 ^a^	77.010 ± 3.350 ^a^	4.884 ± 0.721 ^a^	0.957 ± 0.141 ^ab^	5.908 ± 0.542 ^a^
LBG	F2	11.070 ± 1.151 ^ab^	61.040 ± 3.420 ^b^	4.681 ± 0.873 ^a^	1.130 ± 0.211 ^a^	6.466 ± 0.252 ^a^
(galactomannan)	F3	9.557 ± 0.452 ^b^	61.960 ± 5.150 ^b^	3.504 ± 0.143 ^ab^	0.833 ± 0.034 ^abc^	6.348 ± 0.183 ^a^
	F4	9.511 ± 0.656 ^b^	43.850 ± 2.170 ^c^	2.692 ± 0.628 ^b^	0.647 ± 0.151 ^bc^	2.985 ± 0.113 ^b^
	F5	10.223 ± 0.518 ^a^	54.270 ± 3.040 ^b^	2.223 ± 0.353 ^b^	0.548 ± 0.087 ^c^	2.917 ± 0.576 ^b^
	F1	10.286 ± 0.354 ^a^	17.000 ± 5.370 ^a^	3.268 ± 0.684 ^a^	0.486 ± 0.102 ^a^	6.276 ± 0.083 ^a^
Starch	F2	7.798 ± 0.662 ^b^	16.788 ± 1.304 ^a^	3.139 ± 0.336 ^a^	0.526 ± 0.056 ^a^	3.166 ± 0.197 ^c^
	F3	7.414 ± 0.579 ^b^	11.037 ± 1.711 ^ab^	3.378 ± 0.477 ^a^	0.695 ± 0.098 ^a^	3.670 ± 0.212 ^c^
	F4	6.796 ± 1.079 ^b^	9.341 ± 1.057 ^b^	3.077 ± 1.280 ^a^	0.633 ± 0.263 ^a^	3.092 ± 0.383 ^c^
	F5	7.567 ± 0.369 ^b^	14.830 ± 0.929 ^ab^	2.566 ± 0.580 ^a^	0.638 ± 0.144 ^a^	4.550 ± 0.369 ^b^
	F1	9.597 ± 0.690 ^a^	25.596 ± 1.573 ^a^	3.438 ± 0.925 ^a^	0.603 ± 0.162 ^b^	3.363 ± 0.141 ^a^
LBG/Starch	F2	8.711 ± 0.534 ^ab^	12.015 ± 1.166 ^b^	3.165 ± 0.245 ^ab^	0.572 ± 0.044 ^b^	3.307 ± 0.034 ^a^
(25:75)	F3	8.265 ± 0.087 ^ab^	22.850 ± 5.920 ^a^	2.971 ± 0.191 ^ab^	0.587 ± 0.038 ^b^	3.495 ± 0.705 ^a^
	F4	7.785 ± 0.252 ^b^	14.549 ± 1.597 ^b^	2.123 ± 0.261 ^b^	0.694 ± 0.085 ^b^	3.355 ± 0.946 ^a^
	F5	5.574 ± 0.960 ^c^	9.057 ± 0.371 ^b^	3.580 ± 0.359 ^a^	1.352 ± 0.135 ^a^	2.896 ± 0.765 ^a^

F1 = control film; F2 = control film with added emulsifier; F3 = film with BO as additive; F4 = film with BO as additive/plasticizer (partial glycerol replacement); F5 = film with BO as plasticizer (total glycerol replacement). Means with the same letters in the same column for a specific basis carbohydrate do not show statistical differences (*p* > 0.05).

## Data Availability

The original contributions presented in the study are included in the article/Supplementary Material; further inquiries can be directed at the corresponding author.

## Supplementary Materials

The following supporting information can be downloaded at: https://www.mdpi.com/article/10.3390/foods14244330/s1, Table S1: Normalized areas and corresponding ratios of the bands at 1048 and 1022 cm^−1^ from the FTIR spectra of the films containing starch.

## Abbreviations

The following abbreviations are used in this manuscript:

BO	Buriti oil
DCA	Departamento de Ciência de Alimentos
DEMEC	Departamento de Engenharia Mecânica
EM	Elasticity Modulus
FTIR	Fourier Transform Infrared
EAB	Elongation at Break
LBG	Locust bean gum
LLPDE	Linear low-density polyethylene
MC	Moisture content
OXP	Oxygen permeability
PPGCA	Programa de Pós-Graduação em Ciência de Alimentos
TGA	Thermogravimetric analysis
TS	Tensile strength
WLD	Weight loss derivative
WS	Water solubility
WVP	Water vapor permeability
WVTR	Water vapor transmission rate
