# Supplementary material for: Evaluation of Buriti (Mauritia flexuosa L.) Oil as an Additive for Carbohydrate-Based Biodegradable Films
Source: Foods. 2025 Dec 16;14(24):4330. doi: 10.3390/foods14244330 (PMC12733185; doi:10.3390/foods14244330)
Supplement: Supplementary file 1 [file foods-14-04330-s001.zip › foods-4003662-supplementary.pdf]

Table S1. Normalized areas and corresponding ratios of the bands at 1055 and 1022 cm^{-1} from the FTIR spectra of the films containing starch.

**Sample**		**Band**	**Area**	$R_{1048/1022}$
Starch	**F1**	1021	0.0015000	0.39
		1048	0.0005883	
	**F2**	1022	0.0014700	0.08
		1046	0.0001193	
	**F3**	1025	0.0032300	0.22
		1056	0.0007233	
	**F4**	1025	0.0015900	0.25
		1057	0.0003903	
	**F5**	1024	0.0025300	0.22
		1056	0.0005490	
LBG/Starch (25:75)	**F1**	1021	0.0305000	0.04
		1047	0.0013700	
	**F2**	1022	0.0016900	0.02
		1055	0.0000396	
	**F3**	1022	0.0033400	0.12
		1055	0.0004022	
	**F4**	1024	0.0015900	0.10
		1055	0.0001570	
	**F5**	1025	0.0015500	0.13
		1057	0.0002001	
